# Morphologic, Proliferative, and Cytogenetic Changes during In Vitro Propagation of Cat Adipose Tissue-Derived Mesenchymal Stromal/Stem Cells

**DOI:** 10.3390/ani14162408

**Published:** 2024-08-20

**Authors:** Agustina Algorta, Rody Artigas, Analía Rial, Uruguaysito Benavides, Jacqueline Maisonnave, Kevin Yaneselli

**Affiliations:** 1 Unidad de Inmunología e Inmunoterapia, Departamento de Patobiología, Facultad de Veterinaria, Universidad de la República (UdelaR), Montevideo 13000, Uruguay; ubenavides@gmail.com (U.B.); jacmaiso@gmail.com (J.M.); 2 Unidad de Genética y Mejoramiento Animal, Departamento de Producción Animal y Salud de los Sistemas Productivos, Facultad de Veterinaria, Universidad de la República (UdelaR), Montevideo 13000, Uruguay; rodyartigas@gmail.com; 3 Departamento de Desarrollo Biotecnológico, Instituto de Higiene, Facultad de Medicina, Universidad de la República (UdelaR), Montevideo 11600, Uruguay; analia.rial@gmail.com

**Keywords:** feline, mesenchymal stem cells, cytogenetic, instability, morphology, proliferation

## Abstract

**Simple Summary:**

Stem cell therapy for veterinary patients such as cats often requires a significant quantity of mesenchymal stromal/stem cells (MSCs), typically expanded in laboratory conditions. However, this expansion process can alter the characteristics and genetic stability of these cells. This study focused on assessing the characteristics of MSCs derived from the adipose tissue of cats (cAT-MSCs) during culture conditions. For this purpose, cell morphological features, growth behavior, and cytogenetic stability were examined in passages 2, 4, and 6. Additionally, MSCs’ multipotency and surface markers were assessed. The cAT-MSCs exhibited a spindle-shaped morphology, typical of MSCs. As the cells were cultured over successive passages, a reduction in their growth rate was observed after passage 4, accompanied by abnormalities in the nuclei. cAT-MSCs showed multipotency and their surface marker expression aligned with the expected immunophenotype of MSCs. Cytogenetic studies revealed some structural abnormalities in the chromosomes of the cAT-MSCs, such as gaps, breaks, deletions, duplications, and early chromatid segregation. Nevertheless, these alterations did not show a significant increase over subsequent passages. In conclusion, cAT-MSCs decreased their proliferative capacity after passage 4, accompanied by morphological alterations and signs of structural instability.

**Abstract:**

Stem cell therapy in cat patients needs a high quantity of mesenchymal stromal/stem cells (MSCs) requiring in vitro propagation under culture conditions which may potentially impact cellular characteristics and genetic stability. This study aimed to assess the in vitro characteristics and cytogenetic stability of cat adipose tissue-derived MSCs (cAT-MSCs). For this purpose, morphological features, clonogenic potential, and proliferative capacity of cAT-MSCs were assessed at passages 2 (P_2_), P_4_, and P_6_. Multipotency and immunophenotype were evaluated. Cytogenetic analyses were conducted up to P_6_. The cAT-MSCs exhibited a spindle-shaped morphology in early passages. The doubling time increased from 2.5 days at P_2_ to 9.4 at P_4_ and 10.5 at P_6_, accompanied by the observation of nuclear abnormalities such as cluster formation, karyorrhexis, karyolysis, and a decline in the mitotic index at P_4_. Cells demonstrated multipotency capacity and were CD45−, CD90+, and CD44+. Metaphase analysis at P_2_ and P_4_ revealed some indications of structural instability such as gaps, breaks, deletions, duplications, and early chromatid segregation, but these alterations did not show an increase across passages. In conclusion, cAT-MSCs decreased their proliferative capacity after P_4_, accompanied by morphological alterations and signs of structural instability.

## 1. Introduction

Mesenchymal stromal/stem cell (MSC) therapy has impacted not only human medicine but also the companion animals sector [1]. Cats are veterinary patients who suffer from chronic diseases for which stem cell therapy has been proposed as an alternative approach. Currently, cat adipose tissue-derived MSCs (cAT-MSCs) are most commonly used in clinical applications because they are easily obtained with minimal discomfort and have superior proliferative ability than bone marrow MSCs [2,3]. 

Cat MSC treatments tend to use between 2 and 20 × 10^6^ cells per kilogram of body weight [3], making the in vitro expansion necessary to obtain an appropriate number of cells for therapy. It is reported that cAT-MSCs exhibit a lower proliferative capacity compared to other domestic animal MSCs such as canines and equines [4,5]. Additionally, a decrease in growth rate and syncytial formation during culture has been reported and attributed to the presence of an endogenous feline retrovirus [6]. Consequently, the in vitro propagation of cAT-MSCs obtained from pet cats is more challenging compared to those from other domestic species.

Cells exposed to long-term in vitro conditions may suffer replicative stress, reduction in replicative potential and multipotency, senescence, accumulation of DNA damage, and cytogenetic alterations leading to genomic instability [7,8,9,10]. One of the most relevant safety assessments of MSC therapy is represented by their genomic stability, which can be altered during in vitro culture [7]. The need to assess the cytogenetic stability of human MSCs prior to clinical application has been established by the International Society of Cell Therapy (ISCT). Adult human MSCs seem to maintain cytogenetic stability during in vitro culture compared to embryonic stem cells or induced pluripotent stem cells [11,12,13,14]. Although the majority of the reports show that human MSCs are stable during culture, there is still some controversy regarding genomic stability, and alterations such as aneuploidies have been reported [7,8,15]. Few studies assess the cytogenetic stability of cAT-MSCs for research purposes or before clinical use, analyzing karyotypes in a single passage [16,17,18]. Hence, there is a lack of reports of companion animal MSC chromosomal stability during subsequent passages or analyses on a larger number of metaphases. Furthermore, this is not routinely assessed prior to clinical application in veterinary patients. This study aimed to evaluate the proliferation capacity, multipotency, and cytogenetic stability of cAT-MSCs during in vitro propagation.

## 2. Materials and Methods

### 2.1. cAT-MSC Isolation and Culture

Subcutaneous adipose tissue samples of 0.5–2 g were collected during routine neutering procedures from *n* = 9 young (<1 to 3 years old), clinically healthy, client-owned female cats. A sample of blood (1 mL) without anticoagulant was collected from each donor. Blood samples were centrifuged and serum was used to test for feline immunodeficiency virus (FIV) and feline leukemia virus (FeLV) using FASTest FeLV–FIV (Diagnostik GmbH, Gemeinde Hörbranz Megacor, Austria, cat. Num 751050RG1), and only negative donors were included in this study. Their owners provided informed written consent for research use. 

The adipose tissue sample was washed with phosphate buffer solution (PBS), minced into 1 mm pieces, and digested using 0.1 mg/mL of type I collagenase (Gibco, Grand Island, NY, USA) for 20 min at 37 °C. Growth media (GM) consisting of DMEM (Gibco, NY, USA), 20% fetal bovine serum (FBS; Capricorn, South America; USA), and 1% antibiotic (penicillin G 100 U/mL, streptomycin 100 UI/mL, Capricorn, Düsseldorf, Germany) was added to the digested tissue and then centrifuged at 250× g for 10 min. The obtained pellet (stromal vascular fraction) was resuspended in GM and then seeded in tissue culture plates (Greiner, Kremsmünster, Austria). Plates were incubated at 37 °C and 5% CO_2_ and media were changed every 3–4 days. Once the first adherent cells were observed, FBS was reduced to 10%. When 80% to 90% confluence was attained, the cultures were treated with trypsin-EDTA 0.25% (Sigma Aldrich, SAFC, Buchs, Switzerland) and subcultured.

### 2.2. Morphology

cAT-MSCs were seeded at a density of 10^5^ cells per well at passages 2 (P_2_), P_4_, and P_6_ onto sterile coverslips placed into 6-well plates and cultured for 5 to 7 days in GM. After this period, cells were fixed with 4% paraformaldehyde (diluted with distilled water) for 10 min at room temperature and stained with Giemsa for 15 min. Images were taken with a digital camera (Evolution VF, Media Cybernetics, Rockville, MD, USA) and the software Image Pro-Express 6.0 (Media Cybernetics, MD, USA). Descriptions of nuclear variations on morphology were made according to previous reports [19,20]. 

### 2.3. Colony Forming Units–Fibroblasts (CFU-F)

cAT-MSCs were seeded in triplicate at P_2_, P_4_, and P_6_ at a density of 10^3^ per well in 6-well plates in GM. After 14 days, the colonies formed were fixed with cold methanol (−20 °C) for 10 min and stained with Giemsa for 15 min. Macroscopic colonies were manually counted using ImageJ 1.52a (NIH). A colony was defined macroscopically as a distinct round violet cluster with no less than 50 cells per colony microscopically. The efficiency rate of CFU-F was determined as previously described [21]. 

### 2.4. Doubling Time (DT)

Cells were seeded in triplicate at P_2_, P_4_, and P_6_ at a density of 10^5^ per well in 6-well plates in GM. Cells were cultured to 80% confluence, harvested, stained with trypan blue, and counted in a Neubauer chamber. The DT was determined as previously described [21] (http://www.doubling-time.com/compute.php, accessed on 1 February 2019).

### 2.5. Trilineage Differentiation Assay

Adipogenic, chondrogenic, and osteogenic differentiation assays were performed according to our previous report [22]. Cells were seeded in triplicate at P_2_, P_4_, and P_6_ at a density of 10^4^ per well in 24-well plates with GM. After 48 h, media were changed to induction-specific media. Cells were cultured for 21 days, and the medium was changed every 3–4 days and fixed with 4% paraformaldehyde. The presence of lipid droplets was confirmed by Oil Red O staining (Sigma, St. Louis, MI, USA), the presence of the cartilaginous matrix was confirmed by Alcian Blue staining (Biomedicals, Santa Ana, CA, USA), and the presence of the mineralized matrix was confirmed by Alizarin Red S staining (Biomedicals, Santa Ana, CA, USA). Control cultures were maintained under the same conditions.

### 2.6. Immunophenotype

The MSC surface markers were assessed by flow cytometry as previously reported [22,23]. Briefly, cryopreserved cells were thawed, rapidly washed with PBS, and resuspended in PBS (Mg++ and Ca++ free) containing 5 mM EDTA and 1% FBS (Capricorn Scientific, Ebsdorfergrund, Germany) (FACS EDTA). Then, 10^6^ cells were immunostained with allophycocyanin-conjugated anti-CD44 (clone IM7; Leinco Technologies, Fenton, MO, USA), fluorescein isothiocyanate-conjugated anti-CD45/LCA (CD45RO, clone UCHL1; Acris Antibodies, Herford, Germany), and phycoerythrin-conjugated anti-CD90 (THY1, clone 5E10; antibodies-online, Limerick, PA, USA) antibodies. Then, cells were washed and resuspended in FACS EDTA. At least 20,000 events per sample were acquired on a FACS Canto II system (BD Biosciences, Franklin Lakes, NJ, USA) using FACS DIVA Software V6.1.3 (BD) for acquisition. Cells were gated based on forward/side scatter patterns. Offline analysis was performed using FlowJo software V10.9.0 (BD) (Tree Star, San Carlos, CA, USA).

### 2.7. Cytogenetic Analysis

Cytogenetic stability was evaluated in *n* = 6 cultures at P_2_, P_4_, and P_6_. Cells were seeded in a density of 2 × 10^5^ cells per T25 flask. When the culture reached a confluence of 50–60%, Colcemid (10 mg/mL) was added to each flask to a final dilution of 0.1 μg/mL and then incubated for 2 h at 38 °C. The confluency percentage and time of cell exposure to colcemid were determined by internal assays. 

The cAT-MSCs were harvested using 0.25% trypsin-EDTA. Cells were transferred to a centrifuge tube containing 3 mL of GM. Samples were centrifuged at 250× *g* for 5 min at room temperature. A hypotonic solution consisting of 5 mL of 0.075M KCl was slowly and carefully added to each sample, followed by incubation for 15 min at 38 °C and fixed using a methanol–acetic acid (3:1) solution. The samples were washed three times in a cold solution (5 °C) of methanol–acetic acid (3:1) and stored at 4 °C. Chromosome slides were prepared on the surface of cold methanol (−20 °C) preconditioned slides, then fixed with Bunsen flame, stained with Giemsa 3%, and observed with an optic microscope (Olympus BX60) under 100× magnification. A minimum of 10 metaphases were evaluated per culture. Karyotypes were made according to cat chromosomic standard international nomenclature [24] and alterations were classified according to Udroiu et al. [25]. The mitotic index was calculated as the number of metaphases observed every 1000 nucleated cells and expressed as a percentage. 

### 2.8. Statistical Analysis

Data analysis was performed using GraphPad Prism version 6.01 software (GraphPad Software, Boston, MA, USA). Normality was analyzed by the Kolmogorov–Smirnov test. Proliferative tests, cytogenetic, and morphology analysis were conducted by descriptive analysis, parametric tests (*t*-tests), and non-parametric tests (Mann–Whitney U test or Fisher exact test). Results are expressed as mean ± SD. *p* values < 0.05 were considered statistically significant.

## 3. Results

### 3.1. Cell Morphology and Proliferation

#### 3.1.1. Cell Morphology

**Figure 1:**
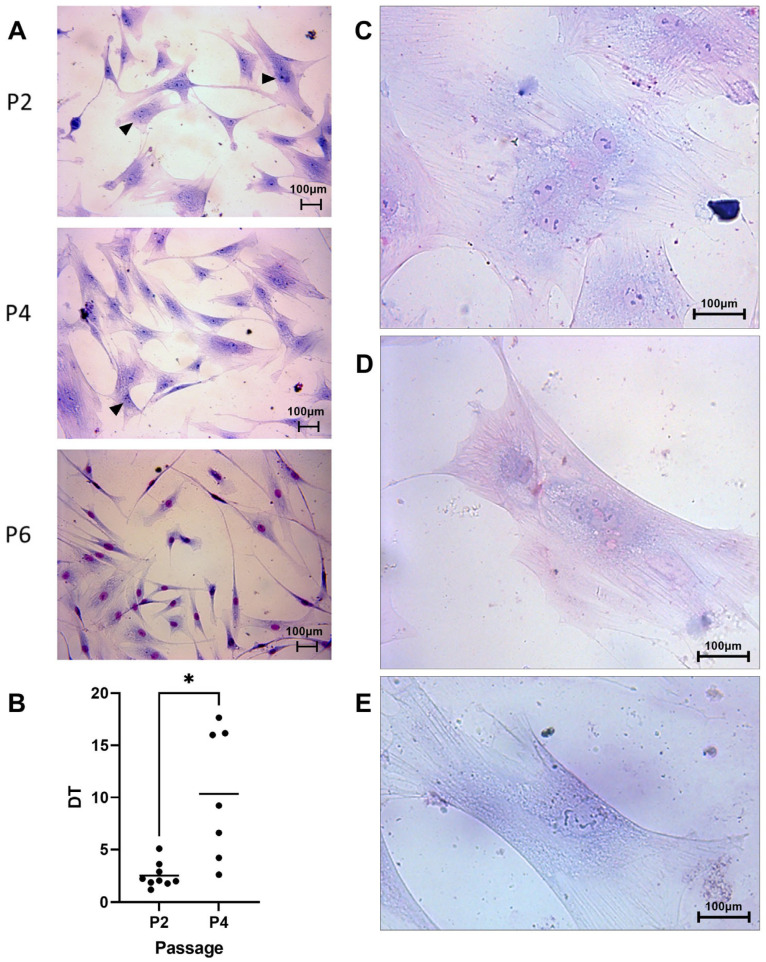
Cell morphology with Giemsa staining. (**A**) Microscopic images (20×) in passages 2, 4, and 6. Similar cell morphology is observed in passages 2, 4, and 6 in this individual. Binucleated cells are indicated with a black arrowhead. (**B**) Doubling time (DT) assay showed an increase in P_4_ (* *p* = 0.01). (**C**) Multinucleated cell with 4 defined nuclei. (**D**) Rectangular-shaped cell with 3 nuclei stained with Giemsa. (**E**) Karyolitic cells, fragmented chromatin, and no defined nuclear membrane are observed.

cAT-MSCs were isolated by the enzymatic method and the first cells were observed 3.7 ± 2.7 days after initial plating. These cells showed a spindle-shaped or triangular morphology and tended to grow homogeneously in a monolayer during P_1_ and P_2_ (Figure 1). 

Some cultures in P_3_ (n = 2/9), P_4_ (n = 2/7), and P_5_ (n = 4/6) showed a notorious reduction in growth rate as well as cluster formation and morphologic changes (Figure 1). 

In P_2_, cells showed a spindle shaped or triangular shape with one defined nucleus and some binucleated cells (Figure 1A). In P_4_, spindle-shaped cells were observed as well as other cultures showing rectangular cells (Figure 1D), bigger cells, and a nuclear pattern that suggested karyorrhexis and a loss in nuclear membrane definition showing karyolysis (Figure 1E). Also, two cultures showed syncytial formations and multinucleated cells (Figure 1C). Finally, P_6_ showed bigger cells with larger cytoplasm prolongations, a rectangular shape (Figure 1D,E), some binucleated cells, and signs of karyorrhexis or karyolysis. 

#### 3.1.2. Cell Proliferation 

Clonogenic capacity: The colony-forming efficiency was 3.6 ± 1.9% in P_2_, 1.8 ± 1.7% in P_4_, and only one sample formed colonies in P_6_ with 5.4% efficiency. No significant differences were observed in clonogenic capacities between P_2_ and P_4_, and P_6_ was not considered for analysis. 

DT assay: The DT increased during passages obtaining 2.5 ± 1.2 days (9/9) in P_2_, 9.4 ± 6.2 days (7/9) in P_4_, and 10.6 ± 11.9 days (2/9) in P_6_. A significant difference was observed between P_2_ and P_4_ (*p* = 0.01) (Figure 1B). Furthermore, when the studied cells in P_4_ were divided into two groups based on morphology using Giemsa staining (5/7), we observed the following behavior. Those cells that maintained a constant growing rate (2/7; DT < 5 days) did show a similar morphology to P_2_. However, cells with higher DT (3/7; DT > 5 days) showed bigger cells, with rectangular morphology, karyorrhexis, and karyolysis signs (Figure 1E).

### 3.2. Characterization of MSCs

#### 3.2.1. Multilineage Differentiation

**Figure 2:**
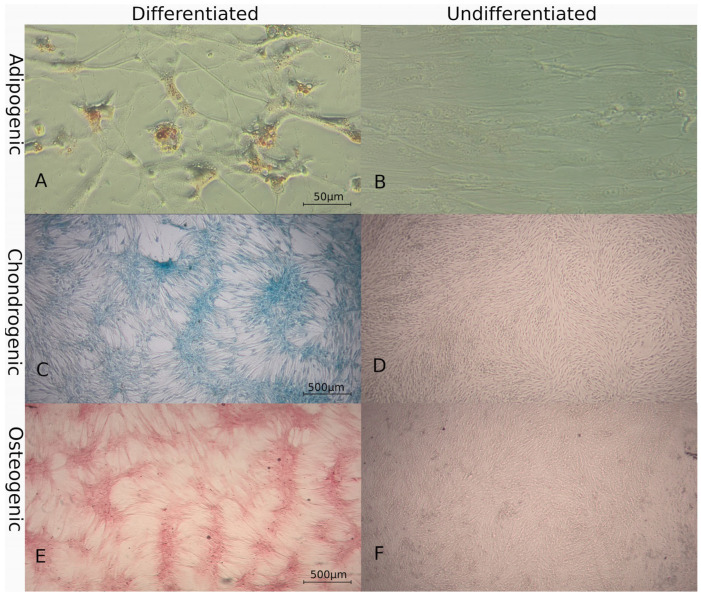
Trilineage differentiation of cat adipose tissue-derived mesenchymal stromal/stem cells in passage 2. Microscopic images of (**A**) adipogenic differentiation stained with Oil Red O showing lipid vacuoles (40×), (**C**) chondrogenic differentiation stained with Alcian blue showing a matrix rich in glycosaminoglycans (10×), and (**E**) osteogenic differentiation stained with Alizarin red showing calcium deposits (10×). (**B**,**D**,**F**) Undifferentiated controls, (**B**) at 40× and (**D**,**F**) at 10×.

Four cultures were used for this assay and successful in vitro trilineage differentiation was achieved. Adipogenic lineage was evidenced by Oil Red O staining of lipidic droplets after 2 weeks under conditioned media (Figure 2A,B). Chondrogenic lineage was observed after 1 week by staining the cartilaginous matrix with Alcian Blue (Figure 2C,D). Osteogenic differentiation was observed after 2 weeks by staining the mineral matrix with Alizarin Red (Figure 2E,F). Control cultures did not show morphologic changes of specific staining affinity.

#### 3.2.2. Immunophenotype

**Figure 3:**
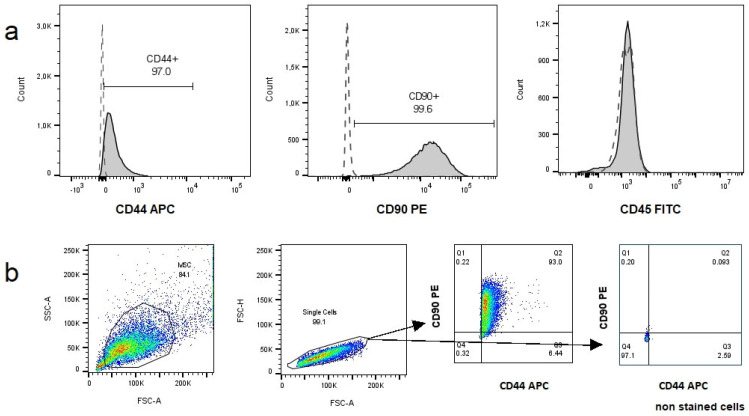
Immunophenotypic analysis of cat adipose tissue-derived mesenchymal stromal/stem cells (cAT-MSCs). (**a**) Representative histograms of CD90, CD44, and CD45 expression of cAT-MSCs (dashed line on each histogram represents non-stained cells). (**b**) Dot plots representing the gating strategy employed: cells were gated based on forward scatter (FSC) and side scatter (SSC) properties, single cells were then selected and CD90 and CD44 expression was analyzed. Representative dot plots of CD44 vs. CD90 expression of cAT-MSCs and non-stained cells are shown. All cAT-MSCs cells were CD45 negative. FITC = fluorescein isothiocyanate; APC = allophycocyanin; PE = phycoerythrin.

Two samples at P_4_ were immunophenotyped. Both samples were CD45 negative and CD90 (99%) and CD44 (97%) positive, as shown in Figure 3. 

### 3.3. Cytogenetic Analysis

**Figure 4:**
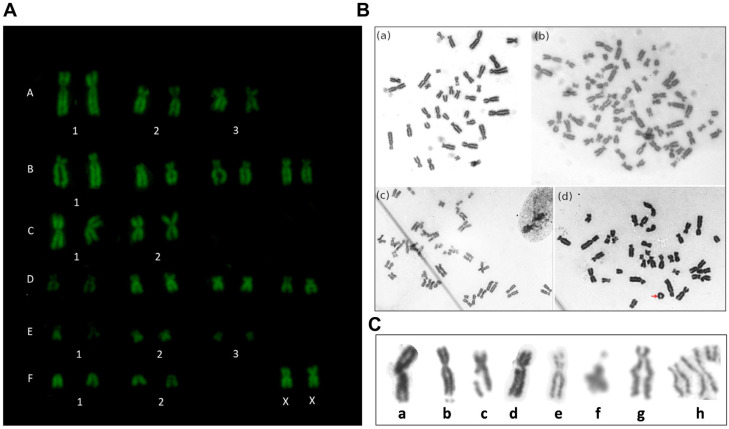
Cytogenetic stability. (**A**) Proposed normal karyotype (2n = 38,XX) stained with Q banding of cat adipose tissue-derived mesenchymal stromal/stem cells (cAT-MSCs) from a female cat. Uppercase letters indicate chromosome groups, and numbers indicate identification within the group. (**B**) Microscopic images (×100 objective) of cAT-MSCs: (**a**) normal metaphase 2n = 38,XX and (**b**) polyploid metaphase 4n = 76,XXXX, (**c**) incomplete polyploid metaphase with diplochromosomes, (**d**) ring chromosome of group F indicated with a red arrow. (**C**) Summary of the main chromosomic structural alterations found in cAT-MSCs which correspond to a. centromeric chromosome fracture, b. chromosome break, c and d. chromatid break, e. gap, f. deletion, g. duplication, h. early chromatid segregations.

Structural and numerical chromosomic aberrations were studied. We analyzed a total of 266 metaphases of six cultures in P_2_ and 61 metaphases of four cultures in P_4_, studying a maximum of 100 and a minimum of 10 metaphases per culture. Three cultures did not show metaphases in P_2_, so they were not considered for the analysis. The mitotic index was 1.1 ± 0.6% for P_2_ and 0.6 ± 0.5% for P_4_ (expressed as mean ± standard error). We did not analyze P_6_ due to an insufficient number of metaphases. The vast majority of the analyzed metaphases exhibited a normal karyotype (Figure 4A), with a few showing numerical alterations. However, structural abnormalities were observed in all cultures. The observed structural aberrations in P_2_ and P_4_ were fractures, gaps, deletions, duplications, and early chromatid segregation, which are summarized in Table 1 and represented in Figure 4C. Small abnormalities may not have been detected due to the detection limits of the applied cytogenetic technique. The chromosomal aberrations observed in this study more frequently affected the chromosomic groups A and B, which are the larger chromosomes in the cat karyotype. 

Polyploidies (4n = 76,XXXX) were observed in P_2_ and diplochromosomes were observed in one culture (Figure 4B). A reduction in the metaphase number was observed in P_4_. Polyploidies were also the most frequent numerical aberration in this passage and a trisomy was observed in one culture (38,XX+1E) which was not observed in P_2_. No significant differences were found between passages in total aberration frequency or between each type of aberration (*p* > 0.1). Three cultures were analyzed in successive passages, observing a decrease in aberration number in subsequent passages, but differences were not statistically significant (*p* > 0.05).

## 4. Discussion

This study evaluated the in vitro characteristics of cAT-MSCs in successive cell passages. Cells with clonogenic capacity and trilineage differentiation ability were isolated. The main findings of this study were that cAT-MSCs showed a reduction in their proliferative capacity accompanied by morphological alterations from P_4_. On the other hand, structural cytogenetic aberrations were observed, although these did not increase in subsequent passages.

Regarding morphology, the first isolated cells were observed 48–72 h after the initial seeding and reached 80% confluence in approximately 7 days. The cell morphology exhibited spindle-shaped or triangular characteristics, in alignment with prior studies [16,26,27]. In subsequent passages, the cells began to adopt a rectangular shape, and binucleated cells were observed after P_4_. This behavior is in accordance with reports in cat bone marrow MSCs [28] as well as other species such as equines [29]. However, other authors found similar morphology in cAT-MSCs until P_5_, observing only minimal alteration up to P_10_ [30], although, these last authors used a different isolation method for cAT-MSCs’ obtention. 

In terms of proliferation dynamics, a significant increase in population DT was observed in P_4_, coinciding with a decrease in clonogenic capacity, although the last was not statistically significant. This aligns with previous reports, where a steady increase in cumulative population DT up to P_4_ or P_5_ and a marked suppression of expansion from P_5_ onwards was observed [31,32]. Other authors also noted that cells up to P_2_ had significantly lower DT than P_3_-P_5_ [21]. In our conditions, cAT-MSCs proliferated adequately until P_4_-P_5_, displaying a lower proliferative capacity compared to other species such as canine [4,33,34] or equine [5] AT-MSCs. This characteristic is disadvantageous when considering cell-based therapies in cats. Additionally, nuclear alterations and a lower mitotic index were observed in the present study. Previous reports [32] evaluated the senescence of cAT-MSCs during culture and found an increase in senescent cells in P_3_, P_5_, and P_7_. Although senescence was not assessed in our study, a morphology consistent with senescent cells was observed from P_4_, suggesting that the use of cAT-MSCs for potential therapeutic purposes should be performed using cells before P_4_.

Furthermore, growth alteration characterized by the formation of cellular aggregates and growth slowdown in P_4_ was observed. Similar behavior was reported [6] in cells obtained from specific pathogen-free (SPF) cats and pet cats, demonstrating that this phenomenon is attributed to the presence and active replication of a clinically insignificant feline retrovirus, Feline Foamy Virus (FFV), leading to syncytium formation in mesenchymal stem cell cultures, impeding their proliferation and inducing apoptosis. In our study, altered morphology was observed in some early-passage cultures, similar to previous report descriptions, suggesting that our donors might have been infected with FFV, although this was not tested in our study.

To characterize cAT-MSCs, we assessed multilineage differentiation and immunophenotype. The studied cells achieved tridifferentiation, confirming their multipotentiality, meeting the characterization standards of animal MSCs [35], and aligning with previous reports for cat MSCs [4,21,31]. We tested two positive surface markers and one negative marker by flow cytometry, obtaining the expected results. However, surface antigens are well defined for human MSCs but not for animal MSCs, especially cats, making the proper selection of surface species-specific antibodies challenging [35,36]. 

This study investigates the in vitro cytogenetic stability at P_2_ and P_4_. A decrease in the number of metaphases was found in P_4,_ aligned with the reduction in their proliferative capacity as mentioned previously. The low number of metaphases obtained in P_6_ coincided with observed morphological alterations, indicative of senescence. The ISCT sets a parameter that 2 identical abnormal metaphases out of 20 analyzed (10%) should be used as the exclusion limit for the clinical use of human MSCs [37]. There are limited precedents regarding the control of cytogenetic stability in cAT-MSCs [16,17,18], which perform cytogenetic control in a single passage but do not conduct continuous monitoring throughout subcultures. In this assay, although some cultures exceeded 20 analyzable metaphases by a considerable margin, others did not reach this quantity, making data analysis challenging. Due to the difficulty in obtaining an acceptable number of metaphases without the use of mitosis stimulants and the decreased proliferation in subsequent passages of cAT-MSCs, cultures with fewer than 20 analyzable metaphases were considered. The analysis was performed on the total number of metaphases per passage, and it was possible to compare three cultures that achieved acceptable metaphases in successive passages. This difficulty in obtaining metaphases has also been reported by other authors for human MSCs [38,39]. The acquisition of sufficient quantities of analyzable metaphases appears to be a weakness in the existing literature, not to mention that studies in cats analyze a limited number of metaphases. It is worth noting that we obtained 100 analyzable metaphases in some individuals, although it should be considered that individual variability factors may also influence these results. Furthermore, there is a lack of previous reports that investigate cAT-MSCs’ stability during in vitro propagation, so more studies on this aspect are needed.

Although signs of chromosomal instability were present, no increase in the number of aberrations was observed between P_2_ and P_4_, suggesting the cytogenetic stability of cells between these passages. Despite the differences were not significant, in the cultures analyzed sequentially, a lower percentage of total chromosomal aberrations was observed in P_4_ compared to P_2_. This has also been reported for human MSCs [39] where a decrease in aberrations from P_3_ and P_5_ onwards was observed. This decrease in aberrations as the passages progress in cell cultures could be explained by an adjustment of MSCs to the culture conditions, with a trend to lose abnormal karyotypes as cell passages increase, possibly through apoptosis or senescence caused by deleterious genetic mutations [39].

In this study, we found that polyploidy was the most common numerical alteration. Previous studies on human gingival mucosa MSCs reported a consistent 3% occurrence of polyploid metaphases across passages [40]. However, we observed that polyploidies occurred in specific cultures and either disappeared or decreased in subsequent passages, supporting the hypothesis of abnormal karyotype loss [39]. 

We observed a monosomic aneuploidy (37,XX-E) in one particular culture in 2 out of 100 metaphases, although this should be confirmed through chromosomal identification techniques to determine if it is a clonal alteration or simply a loss of a chromosome due to the preparation technique. However, the alteration did not persist in the following passage and was considered non-clonal. We also observed a trisomy in P_4_, although we could not confirm if it persisted in subsequent passages; it was considered non-clonal as it was found in only one cell. This differs from findings in human bone marrow MSCs [39] or human MPN-MSCs [41], where aneuploidy was the most frequent alteration. According to the International System for Human Cytogenetic Nomenclature [42], in the case of a loss of a chromosome, it should be present in three cells to be considered clonal. Therefore, in this study, although specific chromosomal identification was not performed, repeated numerical alterations were not observed.

Most studies suggest that MSCs exhibit stable karyotypes [12,43], while others indicate genetic instability or that such instability disappears during culture [39,41], highlighting the controversy surrounding this topic. Specific precedents for cAT-MSCs that suggest cytogenetic stability [16] contradict the findings of our study, although we did not find a concerning increase in numerical alterations in the studied cells. In this study, we used trypsin for cell harvesting and it has been observed that cells harvested using enzymatic methods exhibit a higher number of aberrations than those harvested using mechanical methods [44]. Also, high oxygen percentages or high glucose concentrations can increase chromosomal abnormalities due to oxidative stress [45,46]. On the other hand, cat MSCs are susceptible to FFV infection leading to syncytial formation and impaired proliferation [6]. It is known that cytogenetic instability or the inhibition of DNA repair can be caused by retroviral infections such as Marek’s disease virus, HTLV-1, or BLV [47,48]. The cells used in this study were not tested for FFV. As FFV is an endogenous retrovirus, it could be hypothesized that it might be affecting chromosomal stability, although there are no prior reports to substantiate this hypothesis.

## 5. Conclusions

The cAT-MSCs were isolated using an enzymatic method and exhibited the classical morphology of MSCs in early passages. Their clonogenic capacity was confirmed in three different passages. However, their proliferative capacity declined starting from P_4_, accompanied by morphological alterations. Cells exhibited signs of structural chromosomal instability, but these did not increase between passages. Consequently, cells in early passages may be selected for potential therapies.

## Figures and Tables

**Table 1:** Numerical and structural aberrations observed per passage.

	Aneuploidy	Polyploidy	Fracture	Gap	Deletion	Duplication	Chromatid Separation	Total Number of Aberrations
P2 (n = 266)	3 (1.1%)	13 (4.9%)	18 (6.8%)	5 (1.9%)	6 (2.3%)	9 (3.4%)	9 (3.4%)	63 (23.6%)
P4 (n = 61)	2 (3.3%)	1 (1.6%)	1 (1.6%)	2 (3.3%)	0	2 (3.3%)	4 (6.6%)	12 (19.6%)

## Data Availability

Data supporting the reported results can be sent to anyone interested by contacting the corresponding author.

## References

[1] Hoffman A.M., Dow S.W. (2016). Concise Review: Stem Cell Trials Using Companion Animal Disease Models. Stem Cells.

[2] Quimby J.M., Webb T.L., Habenicht L.M., Dow S.W. (2013). Safety and Efficacy of Intravenous Infusion of Allogeneic Cryopreserved Mesenchymal Stem Cells for Treatment of Chronic Kidney Disease in Cats: Results of Three Sequential Pilot Studies. Stem Cell Res. Ther..

[3] Quimby J.M., Borjesson D.L. (2018). Mesenchymal Stem Cell Therapy in Cats: Current Knowledge and Future Potential. J. Feline Med. Surg..

[4] Voga M., Kovač V., Majdic G. (2021). Comparison of Canine and Feline Adipose-Derived Mesenchymal Stem Cells/Medicinal Signaling Cells With Regard to Cell Surface Marker Expression, Viability, Proliferation, and Differentiation Potential. Front. Vet. Sci..

[5] Vidal M.A., Walker N.J., Napoli E., Borjesson D.L. (2012). Evaluation of Senescence in Mesenchymal Stem Cells Isolated from Equine Bone Marrow, Adipose Tissue, and Umbilical Cord Tissue. Stem Cells Dev..

[6] Arzi B., Kol A., Murphy B., Walker N.J., Wood J.A., Clark K., Verstraete F.J.M., Borjesson D.L. (2015). Feline Foamy Virus Adversely Affects Feline Mesenchymal Stem Cell Culture and Expansion: Implications for Animal Model Development. Stem Cells Dev..

[7] Neri S. (2019). Genetic Stability of Mesenchymal Stromal Cells for Regenerative Medicine Applications: A Fundamental Biosafety Aspect. Int. J. Mol. Sci..

[8] Borgonovo T., Vaz I. (2014). Genetic Evaluation of Mesenchymal Stem Cells by G-Banded Karyotyping in a Cell Technology Center. Rev. Bras. Hematol. Hemoter..

[9] Pan Q., Fouraschen S.M., de Ruiter P.E., Dinjens W.N., Kwekkeboom J., Tilanus H.W., van der Laan L.J. (2014). Detection of Spontaneous Tumorigenic Transformation during Culture Expansion of Human Mesenchymal Stromal Cells. Exp. Biol. Med..

[10] Wang Y., Han Z.-B., Song Y.-P., Han Z.C. (2012). Safety of Mesenchymal Stem Cells for Clinical Application. Stem Cells Int..

[11] Baker D.E.C., Harrison N.J., Maltby E., Smith K., Moore H.D., Shaw P.J., Heath P.R., Holden H., Andrews P.W. (2007). Adaptation to Culture of Human Embryonic Stem Cells and Oncogenesis in Vivo. Nat. Biotechnol..

[12] Bernardo M.E., Zaffaroni N., Novara F., Cometa A.M., Avanzini M.A., Moretta A., Montagna D., Maccario R., Villa R., Daidone M.G. (2007). Human Bone Marrow–Derived Mesenchymal Stem Cells Do Not Undergo Transformation after Long-Term In Vitro Culture and Do Not Exhibit Telomere Maintenance Mechanisms. Cancer Res..

[13] Lamm N., Ben-David U., Golan-Lev T., Storchová Z., Benvenisty N., Kerem B. (2016). Genomic Instability in Human Pluripotent Stem Cells Arises from Replicative Stress and Chromosome Condensation Defects. Cell Stem Cell.

[14] Neri S., Bourin P., Peyrafitte J.-A., Cattini L., Facchini A., Mariani E. (2013). Human Adipose Stromal Cells (ASC) for the Regeneration of Injured Cartilage Display Genetic Stability after In Vitro Culture Expansion. PLoS ONE.

[15] Afonso Cornélio D., Batistuzzo de Medeiros S.R., Cornélio D.A., Medeiros S.R.B. (2014). de Genetic Evaluation of Mesenchymal Stem Cells. Rev. Bras. Hematol. Hemoter..

[16] Clark K.C., Fierro F.A., Ko E.M., Walker N.J., Arzi B., Tepper C.G., Dahlenburg H., Cicchetto A., Kol A., Marsh L. (2017). Human and Feline Adipose-Derived Mesenchymal Stem Cells Have Comparable Phenotype, Immunomodulatory Functions, and Transcriptome. Stem Cell Res. Ther..

[17] Villatoro A.J., Claros S., Fernández V., Alcoholado C., Fariñas F., Moreno A., Becerra J., Andrades J.A. (2018). Safety and Efficacy of the Mesenchymal Stem Cell in Feline Eosinophilic Keratitis Treatment. BMC Vet. Res..

[18] Seo M.S., Kang K.K., Oh S.K., Sung S.E., Kim K.S., Kwon Y.S., Yun S. (2021). Isolation and Characterization of Feline Wharton’s Jelly-Derived Mesenchymal Stem Cells. Vet. Sci..

[19] Thomas P., Holland N., Bolognesi C., Kirsch-Volders M., Bonassi S., Zeiger E., Knasmueller S., Fenech M. (2009). Buccal Micronucleus Cytome Assay. Nat. Protoc..

[20] Bolognesi C., Roggieri P., Ropolo M., Thomas P., Hor M., Fenech M., Nersesyan A., Knasmueller S. (2015). Buccal Micronucleus Cytome Assay: Results of an Intra- and Inter-Laboratory Scoring Comparison. Mutagenesis.

[21] Gómez M.C., Qin Q., Biancardi M.N., Galiguis J., Dumas C., MacLean R.A., Wang G., Pope C.E. (2015). Characterization and Multilineage Differentiation of Domestic and Black-Footed Cat Mesenchymal Stromal/Stem Cells from Abdominal and Subcutaneous Adipose Tissue. Cell. Reprogram..

[22] Algorta A., Artigas R., Rial A., Brandl S., Rodellar C., Benavides U., Maisonnave J., Yaneselli K. (2023). Isolation and Characterization of Feline Dental Pulp Stem Cells. J. Feline Med. Surg..

[23] Yaneselli K., Filomeno A., Semiglia G., Arce C., Erickson K. (2013). Allogeneic Stem Cell Transplantation for Bone Regeneration of a Nonunion Defect in a Canine. Vet. Med. Res. Rep..

[24] Cho K.W., Youn H.Y., Watari T., Tsujimoto H., Hasegawa A., Satoh H. (1997). A Proposed Nomenclature of the Domestic Cat Karyotype. Cytogenet. Cell Genet..

[25] Udroiu I., Sgura A. (2017). Cytogenetic Tests for Animal Production: State of the Art and Perspectives. Anim. Genet..

[26] Webb T.L., Quimby J.M., Dow S.W. (2012). In Vitro Comparison of Feline Bone Marrow-Derived and Adipose Tissue-Derived Mesenchymal Stem Cells. J. Feline Med. Surg..

[27] Martin D.R., Cox N.R., Hathcock T.L., Niemeyer G.P., Baker H.J. (2002). Isolation and Characterization of Multipotential Mesenchymal Stem Cells from Feline Bone Marrow. Exp. Hematol..

[28] Maciel B.B., Rebelatto C.L.K., Brofman P.R.S., Brito H.F.V., Patricio L.F.L., Cruz M.A., Locatelli-Dittrich R. (2014). Morphology and Morphometry of Feline Bone Marrow-Derived Mesenchymal Stem Cells in Culture. Pesqui. Vet. Bras..

[29] Vidal M.A., Kilroy G.E., Johnson J.R., Lopez M.J., Moore R.M., Gimble J.M. (2006). Cell Growth Characteristics and Differentiation Frequency of Adherent Equine Bone Marrow-Derived Mesenchymal Stromal Cells: Adipogenic and Osteogenic Capacity. Vet. Surg..

[30] Panasophonkul S., Samart P., Kongon K., Sathanawongs A. (2017). Phenotypic Characteristics of Feline Adipose-Derived Stem Cells Affected by Cell Passage Number. Pol. J. Vet. Sci..

[31] Kim H.-R., Lee J., Byeon J.S., Gu N.-Y., Lee J., Cho I.-S., Cha S.-H. (2017). Extensive Characterization of Feline Intra-Abdominal Adipose-Derived Mesenchymal Stem Cells. J. Vet. Sci..

[32] LEE B.-Y., LI Q., SONG W.-J., CHAE H.-K., KWEON K., AHN J.-O., YOUN H.-Y. (2018). Altered Properties of Feline Adipose-Derived Mesenchymal Stem Cells during Continuous in vitro cultivation. J. Vet. Med. Sci..

[33] Kang B.-J., Ryu H.-H., Park S.S., Koyama Y., Kikuchi M., Woo H.-M., Kim W.H., Kweon O.-K. (2012). Comparing the Osteogenic Potential of Canine Mesenchymal Stem Cells Derived from Adipose Tissues, Bone Marrow, Umbilical Cord Blood, and Wharton’s Jelly for Treating Bone Defects. J. Vet. Sci..

[34] Yaneselli K.M., Kuhl C.P., Terraciano P.B., de Oliveira F.S., Pizzato S.B., Pazza K., Magrisso A.B., Torman V., Rial A., Moreno M. (2018). Comparison of the Characteristics of Canine Adipose Tissue-Derived Mesenchymal Stem Cells Extracted from Different Sites and at Different Passage Numbers. J. Vet. Sci..

[35] Bourin P., Bunnell B.A., Casteilla L., Dominici M., Katz A.J., March K.L., Redl H., Rubin J.P., Yoshimura K., Gimble J.M. (2013). Stromal Cells from the Adipose Tissue-Derived Stromal Vascular Fraction and Culture Expanded Adipose Tissue-Derived Stromal/Stem Cells: A Joint Statement of the International Federation for Adipose Therapeutics and Science (IFATS) and the International Society for Cellular Therapy (ISCT). Cytotherapy.

[36] Guest D.J., Dudhia J., Smith R.K.W., Roberts S.J., Conzemius M., Innes J.F., Fortier L.A., Meeson R.L. (2022). Position Statement: Minimal Criteria for Reporting Veterinary and Animal Medicine Research for Mesenchymal Stromal/Stem Cells in Orthopedic Applications. Front. Vet. Sci..

[37] Barkholt L., Flory E., Jekerle V., Lucas-samuel S., Ahnert P., Bisset L., Büscher D., Fibbe W., Foussat A., Kwa M. (2013). Risk of Tumorigenicity in Mesenchymal Stromal Cell e Based Therapies—Bridging Scientific Observations and Regulatory Viewpoints. Cytotherapy.

[38] Muntión S., Sánchez-Guijo F.M., Carrancio S., Villarón E., López O., Diez-Campelo M., San Miguel J.F., del Cañizo M.C. (2012). Optimisation of Mesenchymal Stromal Cells Karyotyping Analysis: Implications for Clinical Use. Transfus. Med..

[39] Stultz B.G., McGinnis K., Thompson E.E., Lo Surdo J.L., Bauer S.R., Hursh D.A. (2016). Chromosomal Stability of Mesenchymal Stromal Cells during in Vitro Culture. Cytotherapy.

[40] Nikitina V., Astrelina T., Nugis V., Ostashkin A., Karaseva T., Dobrovolskaya E., Usupzhanova D., Suchkova Y., Lomonosova E., Rodin S. (2018). Clonal Chromosomal and Genomic Instability during Human Multipotent Mesenchymal Stromal Cells Long-Term Culture. PLoS ONE.

[41] Duailibi M.T., Kulikowski L.D., Duailibi S.E., Lipay M.V.N., Melaragno M.I., Ferreira L.M., Vacanti J.P., Yelick P.C. (2012). Cytogenetic Instability of Dental Pulp Stem Cell Lines. J. Mol. Histol..

[42] Simons A., Shaffer L.G., Hastings R.J. (2013). Cytogenetic Nomenclature: Changes in the ISCN 2013 Compared to the 2009 Edition. Cytogenet. Genome Res..

[43] Blázquez-Prunera A., Díez J.M., Gajardo R., Grancha S. (2017). Human Mesenchymal Stem Cells Maintain Their Phenotype, Multipotentiality, and Genetic Stability When Cultured Using a Defined Xeno-Free Human Plasma Fraction. Stem Cell Res. Ther..

[44] Mitalipova M.M., Rao R.R., Hoyer D.M., Johnson J.A., Meisner L.F., Jones K.L., Dalton S., Stice S.L. (2005). Preserving the Genetic Integrity of Human Embryonic Stem Cells. Nat. Biotechnol..

[45] Forsyth N.R., Musio A., Vezzoni P., Simpson A.H.R.W., Noble B.S., McWhir J. (2006). Physiologic Oxygen Enhances Human Embryonic Stem Cell Clonal Recovery and Reduces Chromosomal Abnormalities. Cloning Stem Cells.

[46] Rebuzzini P., Neri T., Mazzini G., Zuccotti M., Redi C.A., Garagna S. (2008). Karyotype Analysis of the Euploid Cell Population of a Mouse Embryonic Stem Cell Line Revealed a High Incidence of Chromosome Abnormalities That Varied during Culture. Cytogenet. Genome Res..

[47] Di Bucchianico S., Giardi M.F., De Marco P., Ottaviano L., Botti D. (2011). Cytogenetic Stability of Chicken T-Cell Line Transformed with Marek’s Disease Virus: Atomic Force Microscope, a New Tool for Investigation. J. Mol. Recognit..

[48] Philpott S.M., Buehring G.C. (1999). Defective DNA Repair in Cells with Human T-Cell Leukemia/Bovine Leukemia Viruses: Role of Tax Gene. J. Natl. Cancer Inst..

